# ﻿*Platyintybia*, a new genus of Apalochrini (Coleoptera, Melyridae, Malachiinae) from China

**DOI:** 10.3897/zookeys.1201.123141

**Published:** 2024-05-14

**Authors:** Zhenhua Liu, Yuqi Wang, Haitian Song, Bi Ding, Zhiqiang Li

**Affiliations:** 1 Guangdong Key Laboratory of Animal Conservation and Resource Utilization, Guangdong Public Laboratory of Wild Animal Conservation and Utilization, Institute of Zoology, Guangdong Academy of Sciences, Guangzhou 510260, China Institute of Zoology, Guangdong Academy of Sciences Guangzhou China; 2 College of Life Science, Shaanxi Normal University, Xi’an, 710062, China Shaanxi Normal University Xi’an China; 3 Fujian Academy of Forestry, Fuzhou, Fujian, 250012, China Fujian Academy of Forestry Fuzhou China

**Keywords:** key to genera, new combination, new species, soft-winged flower beetles, taxonomy

## Abstract

A new genus of malachiine Melyridae, *Platyintybia***gen. nov.**, is described based on several male-specific characters, along with description of its type species, *Platyintybiazhongshanensis***sp. nov.**, from China. A new combination, *Platyintybiasarawakensis* (Champion, 1921), **comb. nov.**, is proposed after examining the type specimen; this species is newly recorded from China. A key to the genera of Chinese Apalochrini is provided for the first time.

## ﻿Introduction

Apalochrini is one of the monophyletic tribes of malachiine Melyridae, characterized by a shortened pedicel, which is almost concealed in the scape. This tribe comprises more than 40 genera from all over the world, with new genera still being described in the past few years (Evers 1987; Tshernyshev 2015a, 2015b, 2016a, 2020a, 2020b, 2020c, 2021a, 2021b, 2021c; Liu et al. 2020, 2021; Tshernyshev and Shcherbakov 2020). The genera of Apalochrini are distinguished by different combinations of characters of the antenna, head, pronotum, elytra, legs, etc., in males (Evers 1987; Tshernyshev 2015b). Members of this tribe mostly inhabit areas close to water bodies, like streams, lakes, salt lakes, and even oceans (Liu et al. 2021). Larvae and adults of Apalochrini are predators or scavengers, feeding on smaller or dead creatures, and sometimes can be collected on flowers (Horne et al. 2000; Liu et al. 2021; Zhenhua Liu pers. obs.).

In China, 12 genera and 43 species of Apalochrini have been recorded so far. The genera are are *Intybia* Pascoe, 1866, *Laius* Guerin-Meneville, 1830, *Myrmecospectra* Motschulsky, 1858, *Protocollops* Evers, 1991, *Troglocollops* Wittmer, 1965, *Dromanthomorphus* Pic, 1921, *Mimapalochrus* Tshernyshev, 2015, *Pectapalochrus* Tshernyshev, 2016, *Apalochrus* Erichson, 1840, *Hadrocnemus* Kraatz, 1895, *Opisthapalochrus* Evers, 1987, and *Spinapalochrus* Pic, 1919 (Major 2007; Tshernyshev 2015a; Tong et al. 2022, 2023; Liu et al. 2023). About half of the species of Apalochrini that occur in China belong to the genus *Intybia*, which is characterized by simple, 5-segmented front tarsi, a dilated scape and antennomere 3 with antennomere 3 highly modified, and a pronotum that is unbeaded along the margin (Liu et al. 2020). Recently, we collected several specimens from Guangdong and Fujian, China. These specimens are similar to *Intybia* but differ in several characters of the male from the current definition of that genus; thus, a new genus is described here.

## ﻿Materials and methods

Materials examined in this study are deposited in the following institutions:

**IZGAS** Institute of Zoology, Guangdong Academy of Sciences, Guangzhou, China

**FAF** Fujian Academy of Forestry, Fuzhou, China

**BMNH**The Natural History Museum, London, United Kingdom

Specimens for dissections were cleared in 5% solution of KOH for about 12 h at room temperature. The abdomen with the aedeagus was transferred to a cavity slide, and the aedeagus was separated from the abdomen using a hooked, fine dissecting needle. Specimens are mounted on Goodrech cards using white emulsion glue, and the genitalia and terminal abdominal segments are preserved in genitalia vials with glycerol.

Layered images of specimens and male genitalia were captured using the Canon 7D DSLR camera mounted on a Wemacro Focus Stacking Rail, with Canon MPE-65 mm macro lens, Mitutoyo 5× and 10× objective lens, and dual-headed flash, with the aid of Helicon Remote (v. 3.9.10 M) and WeMacro Control software. The images were stacked in Helicon Focus v. 8.1.1 software and edited in Photoshop CC 2022.

The morphological terms used in this paper follow Lawrence and Ślipiński (2013). The following standard measurements are used in this study: body length-from apical edge of clypeus to apex of abdomen; pronotal length-median line from anterior margin to posterior margin; pronotal width-maximum width of pronotum; elytral length-from base of scutellum to elytral apex along suture; elytral width-maximum width across the elytra.

## ﻿Taxonomy

### ﻿ Platyintybia

Taxon classificationAnimaliaColeopteraMelyridae

Liu gen. nov.

917AAFBA-D7EE-5B47-9589-868CD6BCD790

https://zoobank.org/FBC2C1DF-3811-41A8-B075-1ED269D208FB

#### Type species.

*Platyintybiazhongshanensis* Liu & Wang sp. nov.

#### Etymology.

The genus name is a combination of the Latin word *platys* and the genus name *Intybia*; *platys* means broad, referring to the modified terminal antennomere in male. Gender feminine.

#### Diagnosis.

This genus can be recognized from other genera of Apalochrini by combination of the following male specific characters: antenna with scape and antennomere 3 dilated and modified, antennomere 11 enlarged and flattened; head with pair of concavities on head between eyes; front tarsi 4-segmented, without comb on tarsomere 2. It can also be separated from *Collops* Erichson, 1840 and some species of *Notointybia* Liu, Ślipiński & Pang, 2020, which also with 4-segmented front tarsi, by having lateral margins of the pronotum without a bead.

#### Description.

Length about 2.7mm.

**Male.** Head and pronotum black, elytra black with pair of large white spots at about basal fourth and pair of smaller white spots at about anterior fourth; antenna yellow with base of scape and terminal segment black; ventral surface mostly black, abdomen orange with lateral areas more or less black. Vestiture comprising double row of dense, short, whitish setae and sparser, longer, black bristles.

Head subtriangular, widest across eyes; vertex with pair of large concavities besides eyes; frons flattened dorsally, moderately constricted in front of eyes. Dorsal surface covered with dense, short, depressed whitish setae, sparser between antennal insertions and absent on concavities. Eyes relatively large, distinctly protruding laterally, finely facetted. Antenna 11-segmented, laterodorsally inserted on frons; scape and antennomere 3 dilated and modified; antennomere 11 flattened and expanded. Maxillary palps with terminal palpomere dilated, cupped, and apical surface depressed; labial palps with terminal palpomere conical.

Pronotum longer than wide, widest at about middle; lateral margins slightly curved, moderately constricted at base, without lateral carina; posterior margin nearly truncate. Disc finely, densely punctate, smoother at middle, posterior area with shallow transverse depression, covered with dense, depressed setae. Prosternum short, with deep incision anteriorly between pronotum and prosternum. Procoxal cavities transverse, continuous at middle, externally open. Procoxae projecting, with protrochantins exposed. Scutellum with visible part subtrapezoidal, posterior margin almost truncate.

Elytra with dorsal surfaces finely and densely punctate, covered with dense, depressed, whitish setae and longer, sparse, black setae posteriorly; epipleura incomplete, extending to abdomen. Meso- and metaventrite without distinct punctuation, covered with dense depressed setae; metaventrite moderately dilated, with short discrimen; metepisternum broad at base, not extending to posterior margin of metaventrite ventrally. Mesocoxae subtriangular, projecting, with exposed trochantins. Metacoxae subtriangular, sharply narrowed laterally. Legs with femora slightly dilated at about basal third; tibiae slender, covered with dense, short setae along inner edge; hind tibiae slightly curved; tarsal formula 4-5-5, with basal tarsomeres slightly prolonged ventrally.

Abdomen with 6-segmented ventrites, freely articulated, gradually narrowed to posterior. Tergite VIII transverse, subtrapezoidal, with pair of anterior struts; sternite VIII nearly divided, weakly connected by membrane at middle (Fig. 4). Aedeagus slender and curved, with apex upwardly curved; endophallus with 1 slender sclerite and a few short sclerites around it.

**Female.** Similar to male in body shape and colouration, but antenna with basal and apical segments simple, head without concavity on vertex, and front tarsi 5-segmented.

#### Distribution.

China (Fujian, Guangdong); Malaysia (Borneo).

### ﻿ Platyintybia zhongshanensis

Taxon classificationAnimaliaColeopteraMelyridae

Liu & Wang sp. nov.

3D5482F4-0D6B-572A-B047-ABBA0CA609ED

https://zoobank.org/DBE66FE1-0C63-4B64-ACFC-2C58C0DEB831

Figs 1 , 3 , 4

#### Etymology.

The species name is derived from Zhongshan, a city of Guangdong Province in South China, where Kongxia, the type locality of the new species, is located.

#### Material examined.

***Holotype***: China–**Guangdong Prov.** • ♂: Zhongshan, Kongxia Village; 22.39510°N, 113.46785°E; 30 May 2023; net sweeping on grasses near stream; Zhenhua Liu leg.; IZGAS COL0001.

#### Diagnosis.

**Figure 1.**
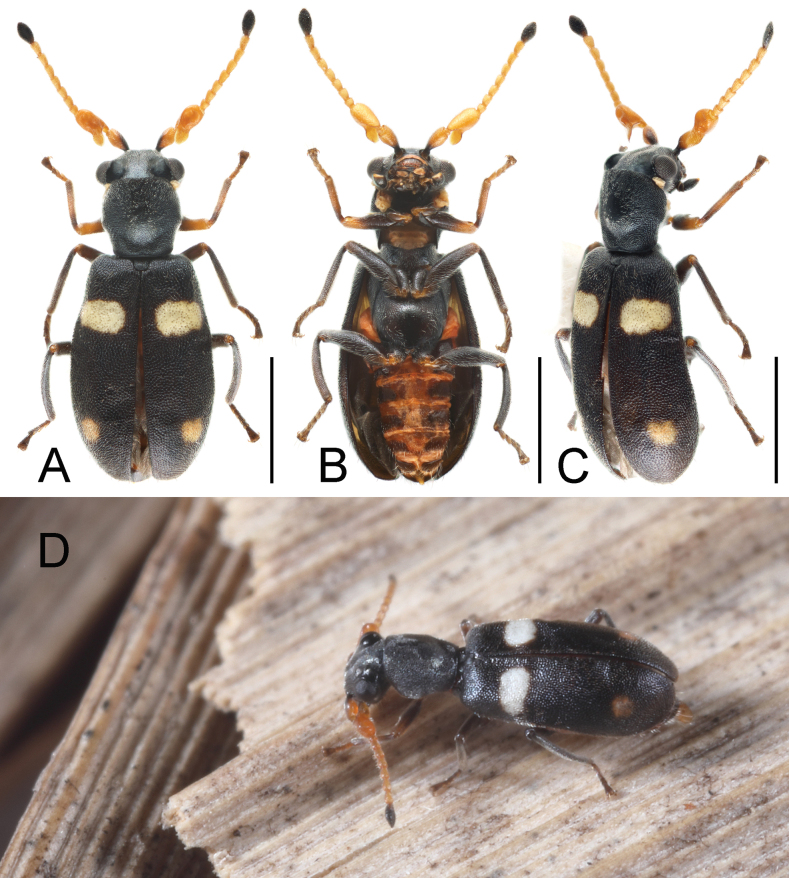
Habitus of *Platyintybiazhongshanensis* Liu & Wang, sp. nov. **A** dorsal view, male **B** ventral view, male **C** lateral view, male **D** habitus photograph. Scale bars: 1 mm for A–C.

The new species resembles *Platyintybiasarawakensis* in the shape of the basal antennomeres and aedeagus, but it can be easily recognized from the latter by the transverse basal spot and a much smaller subapical spot on the elytra (Fig. 1A). It also differs from *P.sarawakensis* in the following characters in males: apical antennomere more rounded (Fig. 3C); front tibiae distinctly slender (Fig. 3D); tergite VIII with posterior margin less emarginate (Fig. 4C); penis less curved laterally (Fig. 4A, F), apex of penis more depressed ventrally (Fig. 4B, G), the shape of long sclerite in inner sac (Fig. 4B, G).

#### Description.

Length 2.7 mm.

**Male.** Antenna mostly yellow, with base and inner edge of scape and apical antennomere black. Legs with middle and hind legs black; front leg with basal half of femora and base of tibiae black, apex of tibiae and apical tarsomere dark brown, remaining parts yellow. Elytra with basal spots whitish and transverse, not extending to lateral or inner suture; subapical spots much smaller, more or less yellowish. Abdominal ventrites mostly orange-red, with lateral areas black.

Head about 1.1 times as wide as pronotum; concavities on head almost extending to anterior margin of eyes; width of vertex between concavities about 1.1 times as wide as concavity across middle of eyes. Dorsal surface of head with pair of circinately arranged setae behind antennal insertions and one on vertex between concavities. Antenna with scape elongate, subtriangular, and constricted at base; antennomere 3 elongate, with a large lamellate process at base, dorsal surface with 1 large, rounded anterior concavity and 2 much smaller basal concavities.

Pronotum about as long as wide. Elytra about 1.6 times as long as wide; epipleura extending to apical margin of elytra but not to apex. Front tibiae about 7.7 times as long as wide, front tarsi 4-segmented.

Abdomen with tergite VIII subtrapezoidal, posterior margin with wide, transverse emargination (Fig. 4C); sternite VIII divided. Male genitalia with penis slender, dorsoventrally curved, apex constricted and upwardly curved; inner sac with a slender and curved sclerite and a few small sclerites around it, apex with dense small denticles (Fig. 4A, B).

**Female.** Unknown.

#### Distribution.

China (Guangdong).

#### Biology.

This species is collected with *Intybiaswatowensis* (Wittmer, 1956) on grass beside a stream in a village, which is consistent with the habitat of most Apalochrini. Feeding habits and behaviour of this species are unknown.

### ﻿ Platyintybia sarawakensis

Taxon classificationAnimaliaColeopteraMelyridae

(Champion, 1921) comb. nov.

F72E506F-2221-59CA-AA73-64FBCAC33E3E

Figs 2 , 3 , 4

Laius sarawakensis Champion, 1921.
Intybia sarawakensis : Plonski 2016: 27.

#### Diagnosis.

As for *Platyintybiazhongshanensis* (Fig. 3F–I).

#### Materials examined.

***Holotype***: Malaysia • ♂: Borneo, Mount Mattang, W. Sarawak, 1000 m elev.; 17 Jan. 1914; G.E. Bryant leg.; BMNH.

#### Other materials examined.

China – **Fujian Province** • ♂: Zhangzhou, Zhangjiangkou mangrove forest; 23°55′21.75″N, 117°24′54.96″E; 17 Oct. 2022; Malaise trap; Rongxiang Su leg.; IZGAS COL0002. • ♀: Zhangzhou, Zhangjiangkou mangrove forest; 23°55′21.75″N, 117°24′54.96″E; 17 Oct. 2022; beating on *Avicenniamarina* (Forsk.) Vierh.; IZGAS COL0003. • 1 ♂, 1 ♀: Zhangzhou, Zhangjiangkou mangrove forest; 23°55′21.75″N, 117°24′54.96″E; 5 m a.s.l.; 29 Aug. 2023; YF Zhang leg.; FAF COL0001 to 0002. • 2 ♀: Zhangzhou, Zhangjiangkou mangrove forest; 23°55′21.75″N, 117°24′54.96″E; 5 m a.s.l.; 16 Sep. 2023; YF Zhang leg.; FAF COL0003 to 0004. • 2 ♂, 5 ♀: Zhangzhou, Zhangjiangkou mangrove forest; 23°55′21.75″N, 117°24′54.96″E; 5 m a.s.l.; 16 Sep. 2023; YF Zhang leg.; FAF COL0005 to 0011.

**Figure 2.**
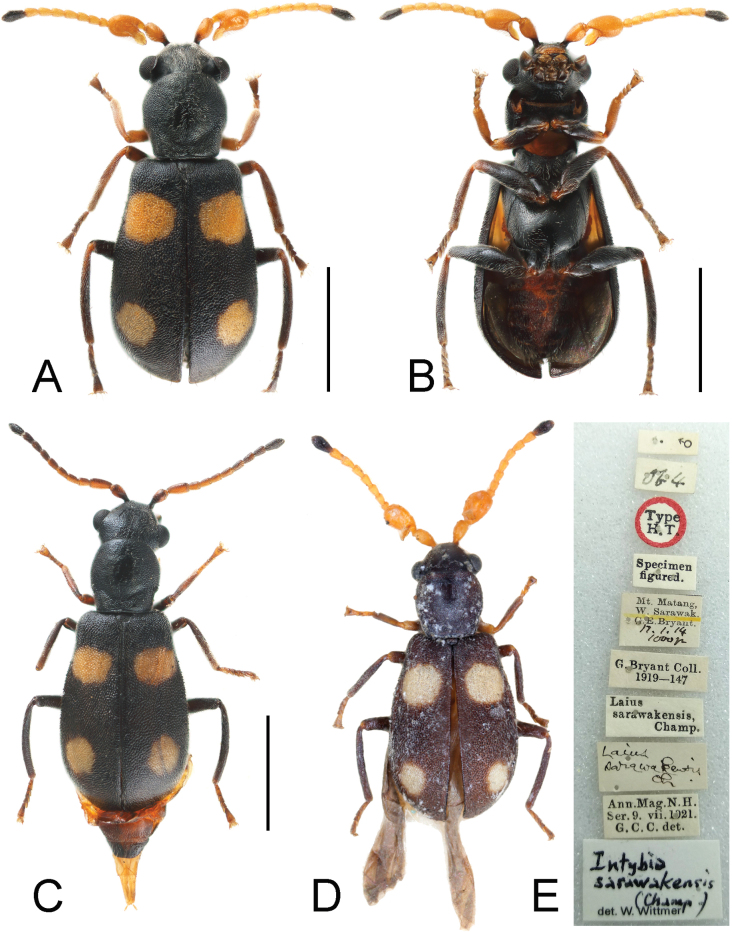
*Platyintybiasarawakensis* (Champion, 1921) **A** dorsal view, male **B** ventral view, male **C** dorsal view, female **D** holotype, male **E** label information. Scale bars: 1 mm (**A–C**).

#### Description.

Length about 2.7 mm.

**Male.** Antenna mostly yellow with base of scape and apical antennomere black. Legs with femora mostly black, apex of front and middle tibiae rufous; front tibiae and tarsi rufous, middle tibiae brownish to black, hind tibiae black with basal part rufous, middle and hind tibiae brownish. Elytra with basal spots large and suboval, not extending to lateral or inner suture; subapical spots rounded, a little smaller. Abdominal ventrites mostly orange-red, with lateral areas black.

**Figure 3.**
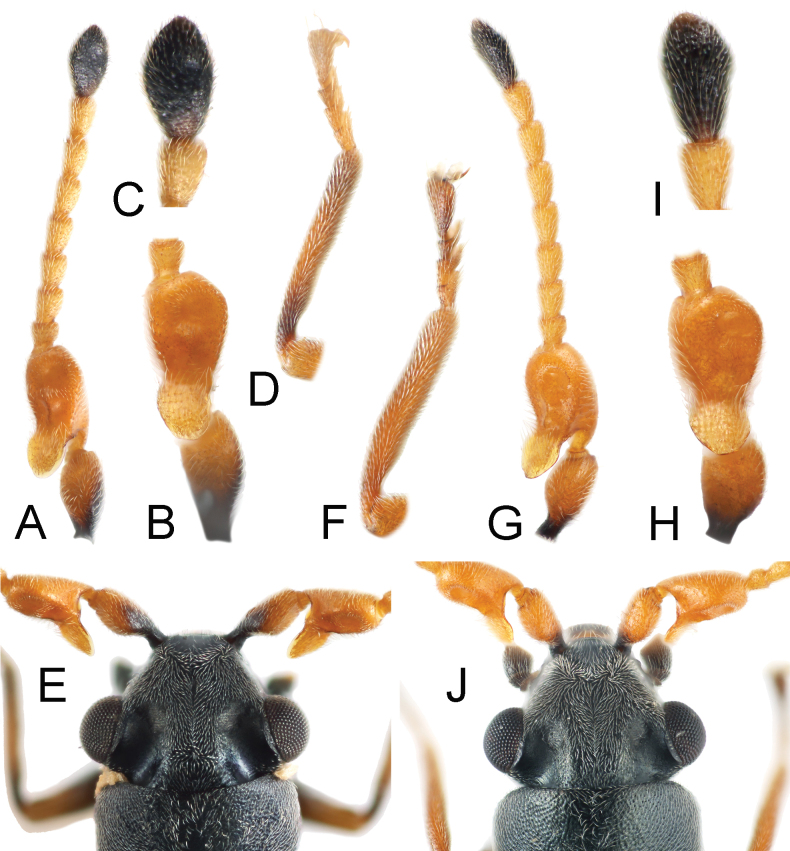
**A–E***Platyintybiazhongshanensis* Liu et Wang, sp. nov. **F–J***Platyintybiasarawakensis* (Champion, 1921) **A, G** antenna, male **B, H** lateral view of basal antennomeres, male **C, I** dorsal view of antennomere 11, male **D, F** fore tibia, male **E, J** dorsal view of head, male.

Head about 1.1 times as wide as pronotum; concavities on head not extending to anterior margin of eyes; width of vertex between concavities about 1.5 times as wide as concavity across middle of eyes. Dorsal surface of head with pair of circinately arranged setae behind antennal insertions. Antenna with scape elongate, subtriangular, and constricted at base; antennomere 3 elongate, with large lamellate process at base, dorsal surface with 1 large, transversely elliptical anterior concavity and 2 indistinct basal concavities.

Pronotum about as long as wide. Elytra about 1.5 times as long as wide; epipleura not extending to apical margin of elytra. Front tibiae about 6.4 times as long as wide, front tarsi 4-segmented.

**Figure 4.**
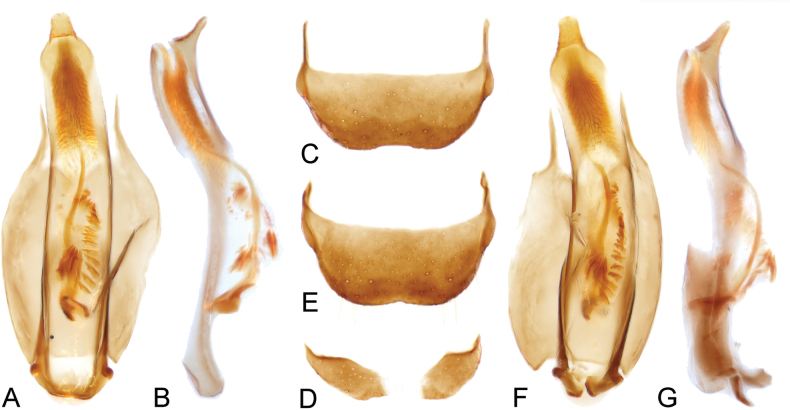
**A–C***Platyintybiazhongshanensis* Liu & Wang, sp. nov. **E–G***Platyintybiasarawakensis* (Champion, 1921) **A, F** male genitalia, dorsal view **B, G** male genitalia, lateral view **C, E** tergite VIII, dorsal view **D** sternite VIII, ventral view.

Abdomen with tergite VIII subtrapezoidal, posterior margin with shallow emargination (Fig. 4E); sternite VIII divided. Male genitalia with penis slender, dorsoventrally curved, apex constricted and upwardly curved; inner sac with a slender, curved sclerite and a few small sclerites around it, apex with dense, small denticles (Figs 4F, 4G).

**Female.** Resembling male in colour and body shape, but with antennae more brownish, scape and antennomere 3 only slightly dilated, terminal antennomere simple; head without concavity on vertex; front tarsi 5-segmented.

#### Distribution.

China (Fujian), Malaysia (Borneo).

#### Biology.

Information on feeding habits and behaviour is scarcely known. The Chinese specimens were found on the leaves of plants in a mangrove forest, and the holotype was collected on Mount Matang without details of the habitat noted.

### ﻿Key to genera of Chinese Apalochrini (males only)

**Table:** 

1	Antenna with scape and antennomere 3 dilated and modified	**2**
–	Antenna with basal antennomeres simple	**7**
2	Front tarsi 4-segmented	***Platyintybia*** gen. nov.
–	Front tarsi 5-segmented	**3**
3	Front tarsi without comb on tarsomere 2	**4**
–	Front tarsi with comb on tarsomere 2	**6**
4	Front tibiae thickened and curved, with concavity at base	***Laius***
–	Front tibiae simple	**5**
5	Body shape ant-like, with prothorax elongated and distinctly constricted posteriorly, elytra constricted at base	***Myrmecospectra***
–	Body shape not ant-like, prothorax never distinctly longer than wide, elytra with humeral area not distinctly constricted	***Intybia***
6	Head with interocular area flat, antennomere 3 dilated and with bunch of hairs	***Protocollops***
–	Head with a deep hollow or protuberance, antennomere 3 dilated but without bunch of hairs	***Troglocollops***
7	Antenna flabellate	**8**
–	Antenna filiform or only expanded	**10**
8	Eyes extremely large, elytra impressed apically	***Mimapalochrus***
–	Eyes not large, elytra simple apically	**9**
9	Vestiture double of white and black setae	***Pectapalcohrus***
–	Vestiture simple of white setae	***Dromanthomorphus***
10	Male-specific character only present on front tarsi, with comb on tarsomere 2	***Apalochrus***
–	Male-specific characters present on antennae, head, tibiae, front trochanter, or abdomen	**11**
11	Abdomen with aculeiform appendage on 4^th^ and 5^th^ sternites	**12**
–	Abdomen without appendage on sternites	***Hadrocnemus***
12	Middle tibiae slightly widened and excavate on inner side	***Opisthapalochrus***
–	Middle tibiae simple	***Spinapalochrus***

## ﻿Discussion

*Platyintybiasarawakensis* was assigned to the *Intybiarouyeri* group mainly on account of its colour pattern of black with two whitish or yellowish spots on each elytron (Plonski 2016). The male-specific characters on the vertex, apical antennomeres, and front tarsi were not mentioned by Champion (1921) when describing this species. More species might be transferred to *Platyintybia* after the holotypes are examined. In Apalochrini, the 4-segmented front tarsi in males have been found in *Collops*, *Notointybia*, and *Platyintybia*, which are distributed in different biogeographic areas (Liu et al. 2020), meaning that this character must have evolved independently in those genera rather than being an autapomorphy. However, relationships between these genera and related genera require further study.

Although *Dromanthomorphus* is included in the key, the only species found in China, *Dromanthomorphusmirabilis* (Pic, 1907) actually lacks some apomorphies, such as excavate front and middle tibiae, swollen metathoracic mesepimera, and possession of an appendage directed forward to the middle coxae (Tshernyshev 2016b; Liu et al. 2023). No nomenclatural act on this species is proposed here, as Isidor Plonski (pers. comm) is working on this species. In the above key, *Dromanthomorphus* specifically refers to *D.mirabilis* only.

## Supplementary Material

XML Treatment for Platyintybia

XML Treatment for Platyintybia zhongshanensis

XML Treatment for Platyintybia sarawakensis

## ﻿Additional information

### Author ORCIDs

Zhenhua Liu https://orcid.org/0000-0002-2739-3305

Yuqi Wang https://orcid.org/0009-0002-0311-8445

Haitian Song https://orcid.org/0000-0003-1042-7959

Bi Ding https://orcid.org/0009-0006-1306-3635

Zhiqiang Li https://orcid.org/0000-0003-0064-7165

### Data availability

All of the data that support the findings of this study are available in the main text.

## References

[B1] Champion GC (1921) Notes on various African and Asiatic species of *Laius*, Guérin, with an account of their accessory ♂-characters [Coleoptera]. Annals and Magazine of Natural History (Series 9) 7(40): 322–343. 10.1080/00222932108632526

[B2] Evers AMJ (1987) Synopsis der Gattung *Apalochrus* Er. und der verwandten Gattungen der Welt (Col., Malachiidae). 63. Beitrag zur Kenntnis der Malachiidae. Annalen Zoologische Wetenschappen 253: 1–73.

[B3] Horne PA Edward CL Kourmouzis T (2000) *Dicranolaiusbellulus* (Gu.rin M.neville) (Coleoptera: Melyridae: Malachiinae), a possible biological control agent of lepidopterous pests in inland Australia. Australian Journal of Entomology 39(1): 47–48. 10.1046/j.1440-6055.2000.00141.x

[B4] Lawrence JF Ślipiński A (2013) Australian Beetles. Vol. 1. Morphology, Classification and Keys. CSIRO Publishing, Collingwood, Victoria, viii + 561 pp. 10.1071/9780643097292

[B5] Liu Z Ślipiński A Pang H (2020) *Notointybia* gen. nov., a new genus of the Australian soft‐winged flower beetles (Coleoptera: Melyridae). Austral Entomology 59(3): 524–534. 10.1111/aen.12481

[B6] Liu Z Ślipiński A Pang H (2021) *Salsolaius* gen. nov. a new genus of Apalochrini (Coleoptera, Melyridae, Malachiinae) from the salt Lake Way of Western Australia. Zootaxa 5082(4): 393–400. 10.11646/zootaxa.5082.4.7 35390956

[B7] Liu Z.H Yu YL Fan CX Xu JZ Yang XK Li ZQ (2023) Seven newly recorded species and genera of Chinese Coleoptera in Haizhu Wetland National Park, Guangzhou. Journal of Environmental Entomology 45(2): 330–341. [In Chinese]

[B8] Major A (2007) Malachiidae. In: Löbl I Smetana A (Eds) Catalogue of Palaearctic Coleoptera, Vol.4. Apollo Books, Stenstrup, 415–455.

[B9] Plonski IS (2016) Studies on the genus *Intybia* Pascoe, 1866 (Coleoptera: Malachiidae) V. Contribution to internal classification and taxonomy, with faunistic and nomenclatorial notes. Zeitschrift der Arbeitsgemeinschaft Österreichischer Entomologen 68: 17–38.

[B10] Tong J Tshernyshev SE Liu H Yang Y (2022) First record of the genus *Troglocollops* (Coleoptera, Malachiidae) from China, with description of a new species. Zootaxa 5195(5): 492–498. 10.11646/zootaxa.5195.5.7 37044411

[B11] Tong J Tshernyshev SE Liu H Yang Y (2023) First record of the genus *Pectapalochrus* Tshernyshev, 2016 (Coleoptera, Malachiidae) from China. Biodiversity Data Journal 11: e104877. 10.3897/BDJ.11.e104877 PMC10848852 38327334

[B12] Tshernyshev SE (2015a) A review of species of the genus *Apalochrus* Erichson (Coleoptera, Malachiidae). Zootaxa 3941(3): 358–374. 10.11646/zootaxa.3941.3.3 25947516

[B13] Tshernyshev SE (2015b) Soft winged flower beetles (Coleoptera: Malachiidae) of the Himalayan region, with notes on the Apalochrini. In: Hartmann M Weipert J (Eds) Biodiversität und Naturausstattung im Himalaya, Volume V.Verein der Freunde & Förderer des Naturkundemuseums, Erfurt, 389–406.

[B14] Tshernyshev SE (2016a) *Pectapalochrus* gen. n., a new genus of soft-winged flower beetles of the tribe Apalochrini (Coleoptera, Malachiidae). Entomological Review 96(3): 348–354. 10.1134/S0013873816030118 34186609

[B15] Tshernyshev SE (2016b) The genus *Dromanthomorphus* Pic, 1921 (Coleoptera, Cleroidea: Malachiidae) in South-East Asia. Zootaxa 4139(4): 551–565. 10.11646/zootaxa.4139.4.7 27470825

[B16] Tshernyshev SE (2020a) *Acroapalochrus* gen. nov.–a new genus of soft-winged flower beetles (Coleoptera: Malachiidae) from West Africa. Journal of Insect Biodiversity 14(1): 001–005. 10.12976/jib/2020.14.1.1

[B17] Tshernyshev SE (2020b) *Mesapalochruspseudorestrictus*, a new genus and species of soft-winged flower beetle of the tribe Apalochrini (Coleoptera: Malachiidae) from Africa. Zoologia Bespozvonocnyh 17(2): 195–201. 10.15298/invertzool.17.2.09

[B18] Tshernyshev SE (2020c) *Protopectinuspseudoparatinus*–a new genus and species of soft winged flower beetle of the tribe Apalochrini (Coleoptera: Malachiidae) from East Africa. Russian Entomological Journal 29(1): 69–72. 10.15298/rusentj.29.1.09

[B19] Tshernyshev SE (2021a) *Dilatapalochrus* gen. nov.-a new species and a new genus of soft-winged flower beetle of the tribe Apalochrini (Coleoptera: Malachiidae) from East Africa. Zootaxa 4966(3): 376–384. 10.11646/zootaxa.4966.3.9 34186609

[B20] Tshernyshev SE (2021b) *Pectotibialispaghmanensis* Tshernyshev gen. nov.–A new genus and species of soft-winged flower beetle (Coleoptera, Cleroidea, Malachiidae) from Afghanistan. European Journal of Taxonomy 775: 1–14. 10.5852/ejt.2021.775.1539

[B21] Tshernyshev SE (2021c) The revision of soft-winged flower beetle genus *Dicranolaius* Champion, 1921 (Coleoptera: Cleroidea: Malachiidae) with description of a new genus *Australolaius* gen. n. from Australia. Zoologia Bespozvonocnyh 18(2): 159–176. 10.15298/invertzool.18.2.07

[B22] Tshernyshev SE Shcherbakov MB (2020) A new genus and species of the soft flower beetles tribe Apalochrini (Coleoptera: Malachiidae) from Africa. Far Eastern Entomologist = Dal’nevostochnyi Entomolog 416: 1–9. 10.25221/fee.416.1

